# Evaluation of improved lung and airway morphology using CT 3D reconstruction after severe scoliosis correction: a retrospective cohort study

**DOI:** 10.1186/s13018-025-06649-4

**Published:** 2026-01-30

**Authors:** Hanwen Zhang

**Affiliations:** Department of Orthopedics, Beijing Jishuitan Hospital, Capital Medical University, 31 Xinjiekou East Street, Xicheng District, Beijing, China

**Keywords:** Severe scoliosis, Posterior spinal fusion, Airway morphology, Lung volume, 3D CT reconstruction, Bronchial dilation

## Abstract

**Background:**

Previous studies have not compared lung and airway morphologies before and after treatment for severe scoliosis. Herein, computed tomography (CT) 3-dimensional (3D) reconstruction of the respiratory system was performed to evaluate the changes in lung expansion and airway dilation following posterior spinal fusion for severe scoliosis.

**Methods:**

Twenty-two patients with severe scoliosis (12 women and 10 men) who treated with posterior spinal fusion (PSF) were included. Pre- and postoperative visits included CT scans and radiographic assessments. Changes in airway dilation were assessed using 3D CT reconstruction of the bronchial tree*,* and changes in lung volume were evaluated using lung 3D CT reconstruction pre- and postoperatively.

**Results:**

Spinal radiographic parameters improved after PSF. The main Cobb angles were 135.89 ± 21.80° and 50.51 ± 16.02° (*p* < 0.001), and thoracic kyphosis angles were 115.75 ± 35.47° and 47.60 ± 8.56° in the pre- and postoperative groups, respectively (*p* < 0.001). After PSF, the total lung volume (TLV) values improved from 1.48 ± 0.76L to 1.89 ± 0.33 L, which were significantly different (t = 3.099, *p* = 0.0101). The preoperative and postoperative values for the left lung volume (0.69 ± 0.34 and 0.88 ± 0.33L) and right lung volume (0.69 ± 0.14 and 0.95 ± 0.28L) were significantly different [t = 3.802; *p* = 0.0029]; [t = 3.415; *p* = 0.0066]. The tracheal diameter varied between the pre- and postoperative groups (12.21 ± 1.91 mm vs. 15.32 ± 1.83 mm, respectively) (*p* < 0.0001). The diameter of the left main bronchus increased from 9.83 ± 2.41 mm to 12.01 ± 1.76 mm (*p* = 0.0138). The diameter of the right major bronchus increased from 9.88 ± 2.41 to 11.63 ± 1.37 mm (*p* = 0.0048).

**Conclusions:**

Lung morphology parameters, including lobe volume and bronchial parameters, can be measured using CT-based reconstructions. PSF can safely and effectively correct the curve, relieve airway obstruction, and expand the lungs of patients with severe scoliosis.

**Level of Evidence III:**

This journal requires that authors assign a level of evidence to each article. For a full description of these Evidence-Based Medicine ratings, please refer to the Table of Contents or the online  Instructions to Authors  www.springer.com/00266.

## Background

Correction of severe scoliosis is particularly challenging when the spinal deformity involves a Cobb angle greater than 100°, which is classified as severe [1–3]. The risk of postoperative pulmonary complications increases with declining pulmonary function [4–6]. Although restrictive lung deficits are common in patients with scoliosis, approximately 46% exhibit obstructive or mixed ventilatory defects preoperatively [7–9].

Lung 3D reconstruction helps reliably estimate the lung volume (LV) in patients with severe scoliosis; the lung can be divided into lobes [10–13]. Researchers have quantified the bronchial diameter and length using 3D imaging, [14–16], facilitating the reconstruction of the bronchial tree [17–19]. Further investigation of pre- and postoperative lung and airway morphologies in patients with severe scoliosis is warranted, driven by advancements in 3D lung and pulmonary bronchial tree reconstruction.

However, no studies have yet evaluated pre- and postoperative airway and lung volume morphological features in patients with severe scoliosis. This study utilized low-dose CT before and after posterior spinal fusion to evaluate the alterations in lung volume and airway parameters. We sought to assess whether the treatment of severe scoliosis affects lung and airway morphological features.

## Materials and methods

### Patient population

This retrospective study was approved by the Institutional Ethics Committee of Beijing Jishuitan Hospital in accordance with the Declaration of Helsinki. All patients provided written informed consent. Thirty-four patients with severe scoliosis who underwent posterior spinal fusion between January 2023 and January 2025 were reviewed. The inclusion criteria were as follows: (1) severe kyphoscoliosis (coronal Cobb angle or thoracic kyphosis angle ≥ 100°) and (2) posterior fusion surgery. The exclusion criteria were as follows: (1) spinal surgery prior to this study and (2) a history of surgery involving the thoracic cage. Demographic parameters included body mass index (BMI), etiology, sex, and age.

### Radiographic parameters

Standing full long spinal X-ray in the posteroanterior and sagittal planes was acquired pre- and postoperatively. Alterations in spinal parameters were also evaluated, including the main thoracic Cobb angle, T1 to T12 height, C7 to S1 height, and kyphosis measurements from T5 to T12.

### Airway reconstruction parameters

CT scans were performed for all patients using the Siemens SOMATOM Sensation 16 system (Germany, slice thickness:1.0 mm), which were used for preoperativeplanning and postoperative assessment of screw trajectories. Data were obtained in DICOM format and subsequently loaded into Mimics Medical 21.0 (Materialise NV, Leuven, Belgium). The segmented airway tool enabled semi-automatic segmentation of the airway tract, with user-initiated segmentation marking the onset of the trachea [14]. The diameter and lumen area of the trachea (superior to the carina) and the right and left major bronchi were measured.

### Lung reconstruction parameters

The Segment Lung Lobes tool detects and fits freeform surfaces through the lung lobe, thereby separating fissures. The freeform surfaces can be updated by adjusting the points. Finally, the lungs were segmented using a freeform surface. The total lung volume (TLV), volume of each lobes, convex-side LV, and concave-side LV were observed on pre- and post-traction CT images using 3D reconstruction, as described in a previous study [20].

### Statistical analysis

Radiographic measurements and airway and lung morphological measurements were compared pre- and postoperatively. Differences were assessed using two-tailed paired Student’s t-tests, and correlations were evaluated using Spearman’s rank test. SPSS version 25.0 (IBM Corp., Armonk, NY, USA) was used for statistical analyses. Statistical significance was set at *p* < 0.05.

## Results

Twenty-two patients with severe scoliosis were analyzed (12 women and 10 men). The mean age was 23.58 ± 6.22 years, and the mean BMI was 15.24 ± 2.18 kg/m^2^. Etiological diagnoses were congenital scoliosis (n = 7), adult idiopathic scoliosis (n = 8), and neuromuscular scoliosis (n = 7). All patients underwent posterior spinal fusion without spinal osteotomy, and none of them had pulmonary complications or required postoperative ventilator support.

After PSF, all spinal radiographic parameters improved. The main Cobb angles were 135.89 ± 21.80° and 50.51 ± 16.02° in the pre-and postoperative groups, respectively (*p* < 0.001). Additionally, the thoracic kyphosis angles were 115.75 ± 35.47° and 47.60 ± 8.56° in the pre-and postoperative groups, respectively (*p* < 0.001). The distances from C7 to S1 were 282.56 ± 40.58 mm and 403.45 ± 40.89 mm in the pre-and postoperative groups, respectively (*p* < 0.001). Furthermore, the T1–T12 distances were 139.14 ± 29.11 mm and 218.25 ± 35.47 mm in the pre-and postoperative groups, respectively (*p* < 0.001) (Table 1).

**Table 1:** Comparison of radiographic parameters between the pre- and post-operative groups

	Pre-operative	Post-operative	*P*-value
Main cobb angle (°)	135.89 ± 21.80	50.51 ± 16.02	< 0.001
C7–S1 (mm)	282.56 ± 40.58	403.45 ± 40.89	< 0.001
T1–T12 (mm)	139.14 ± 29.11	218.25 ± 35.47	< 0.001
Thoracic kyphosis (°)	115.75 ± 35.47	47.60 ± 8.56	< 0.001

### Morphological parameters

The pre- and postoperative TLV values (1.48 ± 0.76 and 1.89 ± 0.33 L, respectively) were significantly different (t = 3.099, *p* = 0.0101). The pre- and postoperative values for the left LV (0.69 ± 0.34 and 0.88 ± 0.33 L) and right LV (0.69 ± 0.14 and 0.95 ± 0.28 L), respectively ([t = 3.802; *p* = 0.0029]; [t = 3.415; *p* = 0.0066]), were significantly different. For further analysis, the lungs were divided into five lobes. The right upper lobe (0.33 ± 0.25 and 0.37 ± 0.19 L, t = 2.159; *p* = 0.0599) and right middle lobe (0.18 ± 0.11 and 0.26 ± 0.12 L, t = 2.020; *p* = 0.0710) did not change significantly after PSF. The right lower lobe (0.29 ± 0.12 and 0.37 ± 0.16 L, t = 2.376; *p* = 0.0421) changed significantly after PSF. Interestingly, the right upper lobe combined with the right middle lobe volume changed significantly after PSF (0.47 ± 0.20 and 0.63 ± 0.23 L, t = 2.613; *p* = 0.0241). The pre and postoperative values for the left upper lobe volume (0.41 ± 0.19 L and 0.56 ± 0.21 L, respectively) and left lower lobe volume (0.22 ± 0.16 L and 0.33 ± 0.15 L, respectively) were significantly different ([t = 3.267, *p* = 0.0075] and [t = 4.317, *p* = 0.0012], respectively). After PSF, the main trachea, left main bronchus, and right main bronchus were dilated. The tracheal diameter exhibited a significant difference between the preoperative and postoperative groups (12.21 ± 1.91 mm vs. 15.32 ± 1.83 mm; *p* < 0.0001). The left main bronchus increased from 9.83 ± 2.41 to 12.01 ± 1.76 mm (*p* = 0.0124). The right major bronchus increased in diameter from 9.88 ± 2.41 to 11.63 ± 1.37 mm (*p* = 0.0058).

**Fig. 1:**
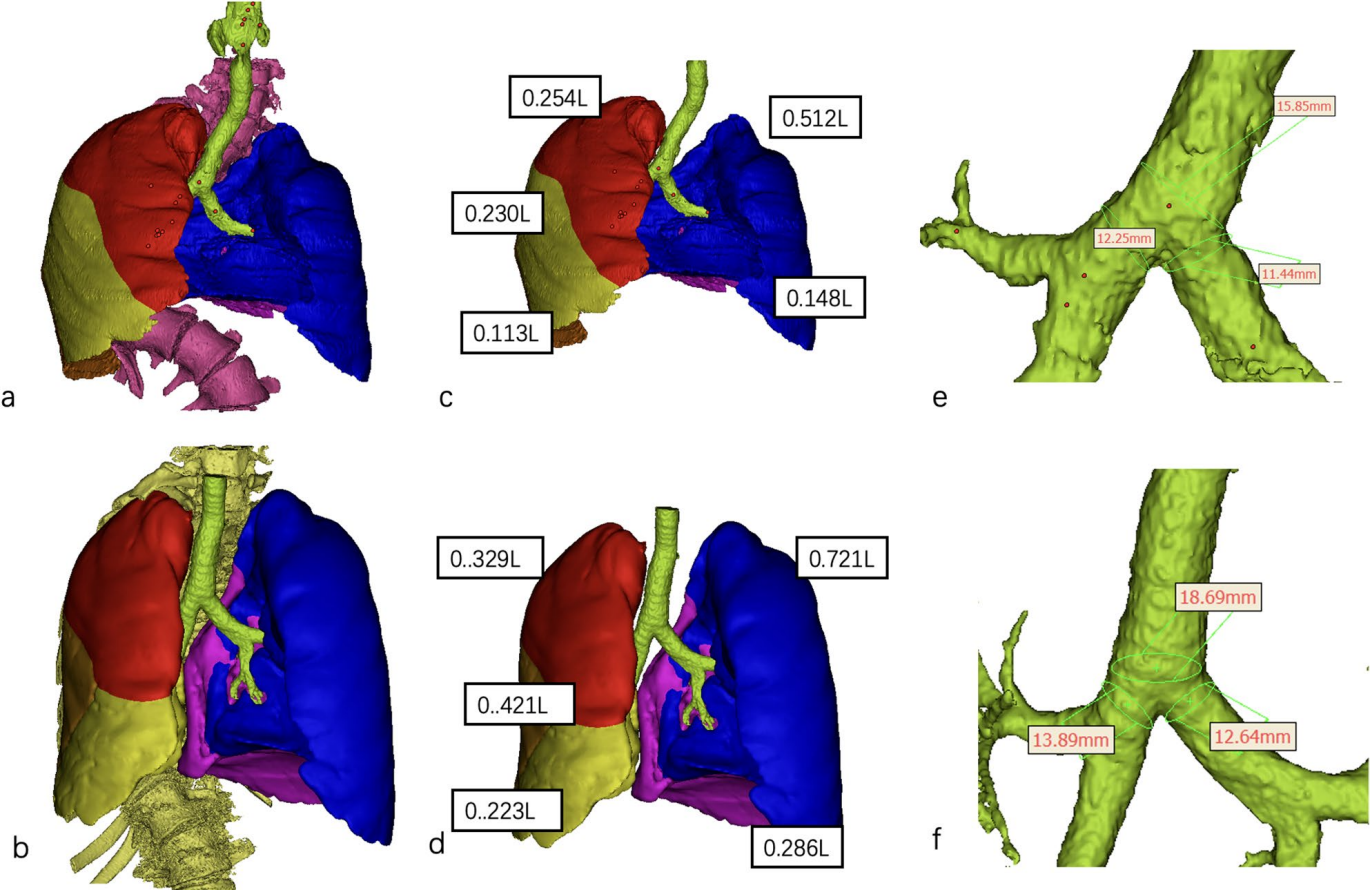
Images of a 28-year-old man with severe scoliosis who underwent posterior spinal fusion. The lobe volume, main trachea, and diameters of the left and right main bronchi improved after posterior spinal fusion. **a** Preoperative 3D-reconstruction of the lung, trachea, and spine. **b** Postoperative 3D-reconstruction of the lung, trachea, and spine. **c** Preoperative 3D-reconstruction of each lobe. **d** Postoperative 3D-reconstruction of each lobe. **e** Preoperative 3D-reconstruction of the bronchial tree. **f** Postoperative 3D-reconstruction of the bronchial tree

The pre- and postoperative groups demonstrated different lumen areas of the trachea (132.11 ± 34.91 mm^2^ vs. 182.41 ± 43.51 mm^2^, *p* < 0.0001), right main bronchi (81.43 ± 32.61 mm^2^ vs. 107.11 ± 24.71 mm^2^, *p* = 0.0078), and left main bronchi (79.26 ± 31.41 mm^2^ vs. 98.34 ± 30.11 mm^2^, *p* = 0.0242) (Table 2). Figure 1 illustrates a representative case.

**Table 2:** Comparison of morphological parameters between the pre- and post-operative groups

	Pre-operative	Post-operative	*P*-value
Total lung volume	1.48 ± 0.76	1.89 ± 0.33	0.0101
Left lung volume	0.69 ± 0.34	0.88 ± 0.33	0.0029
Right lung volume	0.69 ± 0.14	0.95 ± 0.28	0.0066
Right upper lobe volume	0.33 ± 0.25	0.37 ± 0.19	0.0599
Right middle lobe volume	0.18 ± 0.11	0.26 ± 0.12	0.0710
Right lower lobe volume	0.29 ± 0.12	0.37 ± 0.16	0.0421
Right upper lobe combined right middle lobe volume	0.47 ± 0.20	0.63 ± 0.23	0.0241
Left upper lobe volume	0.41 ± 0.19	0.56 ± 0.21	0.0075
Left lower lobe volume	0.22 ± 0.16	0.33 ± 0.15	0.0012
Diameter of trachea	12.21 ± 1.91	15.32 ± 1.83	< 0.0001
Lumen area of trachea	132.11 ± 34.91	182.41 ± 43.51	< 0.0001
Diameter of left main bronchi	9.83 ± 2.41	12.01 ± 1.76	0.0124
Lumen area of left main bronchi	79.26 ± 31.41	98.34 ± 30.11	0.0242
Diameter of right main bronchi	9.88 ± 2.41	11.63 ± 1.37	0.0058
Lumen area of right main bronchi	81.43 ± 32.61	107.11 ± 24.71	0.0078

The main thoracic Cobb angle, thoracic kyphosis, T1–T12 distance, and C7–S1 distance were correlated with TLV. The right LV was correlated with the main thoracic Cobb angle, thoracic kyphosis, and C7–S1 distance, but not with the T1–T12 distance. Additionally, the right upper lobe volume correlated with the T11–T12 and C7–S1 distances. The right middle lobe volume was correlated with the C7–S1 distance, whereas the right lower lobe volume was correlated with the main thoracic Cobb angle. The combined right upper and middle lobe volumes correlated with the main thoracic Cobb angle, thoracic kyphosis, T1–T12 distance, and C7–S1 distance. The left upper and lower lobe volumes were correlated with the main thoracic Cobb angle, thoracic kyphosis, T1–T12 distance, and C7–S1 distance. Thoracic kyphosis was also correlated with the diameters of the trachea, left main bronchus, and right main bronchus (Table 3).

**Table 3:** Correlations between lung morphological parameter and radiological variables

Variable	Main thoracic Cobb angle		TK		T1-T12		C7-S1	
	*P*-value	R coefficients	*P*-value	R coefficients	*P*-value	R coefficients	*P*-value	R coefficients
Total lung volume	0.0009	− 0.6549	0.0078	− 0.5513	0.0127	0.5221	0.0085	0.5462
Right lung volume	0.0041	− 0.5866	0.0284	− 0.4672	0.0949	0.3649	0.0356	0.4501
Left lung volume	0.0063	− 0.5639	0.0170	− 0.5030	0.0069	0.5586	0.0148	0.5122
Right upper lobe volume	0.0768	− 0.3680	0.1019	− 0.3420	0.0489	0.4061	0.0277	0.4490
Right middle lobe volume	0.1196	− 0.3264	0.0734	− 0.3721	0.0764	0.3685	0.0323	0.4380
Right lower lobe volume	0.0446	− 0.4320	0.1834	− 0.2945	0.3590	0.2055	0.1471	0.3196
Right upper lobe combined right middle lobe volume	0.0018	− 0.6272	0.0250	− 0.4763	0.0430	0.4350	0.0151	0.5110
Left upper lobe volume	0.0112	− 0.5299	0.0303	− 0.4623	0.0031	0.6002	0.0083	0.5479
Left lower lobe volume	0.0007	− 0.6683	0.0016	− 0.6308	0.0165	0.5052	0.0044	0.5827
Diameter of trachea	0.2058	− 0.2807	0.0121	− 0.5250	0.2346	0.2643	0.0719	0.3911
Diameter of Left main bronchi	0.4062	− 0.1864	0.0318	− 0.4587	0.3596	0.2052	0.1107	0.3496
Diameter of right main bronchi	0.1175	− 0.3435	0.0020	− 0.6223	0.0776	0.3841	0.0274	0.4697

## Discussion

Severe scoliosis negatively affects pulmonary function, potentially leading to significant morbidity and mortality [21, 22]. Restrictive ventilatory dysfunction is defined as reduced LV, which constrains respiratory capacity and results in decreased oxygen saturation. In contrast, obstructive ventilatory dysfunction is marked by constricted airway diameters and increased airway resistance [23, 24]. Previous studies have estimated pulmonary function based on anterior–posterior and lateral radiographic deformities [25–27]. However, scoliosis is a complex 3D spinal and rib cage deformity, and 2D images provide only limited information. The CT scan method is useful for accurately measuring LV in patients with deformities [11, 12]. PSF not only provides a strong corrective force but also has the potential to maintain or even improve pulmonary function [28, 29]. In this study, TLV, as well as the right and left LVs, increased significantly after PSF. Notably, CT reconstruction allows separation of the right and left lungs and further segmentation into five distinct lobes. The volumes of the right upper, left upper, and left lower lobes increased significantly after PSF. Although the volumes of the right upper and middle lobes did not individually change significantly, their combined volumes showed a significant increase.

Pulmonary complications are common because congenital spine deformities decrease not only the volume but also the function of the lungs [30]. Severe scoliosis significantly affects respiratory function. This effect is most often restrictive owing to severe anatomical distortion of the chest, leading to reduced LV, limited diaphragmatic excursion, and chest wall muscle inefficiency. Bronchial compression by a deformed spine may also occur but is rarer [31]. This study successfully reconstructed pre- and postoperative three-dimensional bronchial trees. Morphological metrics, such as the diameter and lumen area of the left and right main bronchi, were increased following posterior corrective surgery.

Various previous studies have reported conflicting results, ranging from mild improvement to worsening of the condition [32–34]. In this study, the total lung and lobe volumes, as well as the diameter of the central bronchi, improved after PSF. Based on the available evidence, it is reasonable to state that the main goal of scoliosis surgery is to preserve or improve lung function.

The Cobb angle is commonly used to describe the geometric severity of scoliosis. It is approximately correlated with overall clinical severity [35–38], although the exact relationship between scoliosis and lung function is highly complex [31]. Although these problems are more common or severe among patients with severe scoliosis, there is no direct correlation between them and the degree of scoliosis [25, 32, 39–41]. In this study, the main thoracic Cobb angle was correlated with LV parameters. As the Cobb angle was corrected using PSF, the volumes of all five lobes increased. However, the lumbar area and bronchial tree diameter did not correlate with the main thoracic Cobb angle. Prior research has shown a reduction in kyphosis in patients with thoracic scoliosis, which is characterized by spinal encroachment into the thoracic cavity, constricting the airway [8, 25]. Therefore, hypokyphosis is considered a relevant factor for airway obstruction. In this study, thoracic kyphosis was correlated with airway parameters. As kyphosis decreases with PSF, the bronchial airway tree expands. However, lobe volume did not correlate with thoracic kyphosis.

Although these findings offer insights into the anatomical and functional effects of PSF in severe scoliosis, certain limitations must be acknowledged. First, the small sample size may have prevented some comparisons from reaching statistical significance. Second, pulmonary function tests are considered the gold standard for measuring pulmonary impairment. This study only provided static morphological data. Further studies should focus on the relationship between static morphological data and functional data pre- and postoperatively. Various factors such as age, gender, and etiology may affect pulmonary function changes in severe scoliosis after PSF. Large sample size and detailed supplement information are necessary in further studies.

In conclusion, 3D CT reconstruction could be a surrogate method to quantify pulmonary morphological parameters in patients with severe scoliosis. PSF may effectively correct the curve, relieve airway obstruction, and expand the lungs of patients with severe scoliosis.

## Data Availability

Available after consent of Correspondence author for reasonable demand.

## Abbreviations

BMI: Body mass index
CT: Computed tomography
LV: Lung volume
PSF: Posterior spinal fusion
TLV: Total lung volume

## References

[1] Chang KW. Cantilever bending technique for treatment of large and rigid scoliosis. Spine (Phila Pa 1976). 2003;28(21):2452–8. 14595163 10.1097/01.BRS.0000092063.63315.D5

[2] Kandwal P, Vijayaraghavan GP, Nagaraja UB, Jayaswal A. Severe rigid scoliosis: review of management strategies and role of spinal osteotomies. Asian Spine J. 2017;11(3):494–503. 28670419 10.4184/asj.2017.11.3.494 PMC5481606

[3] Balikci T, Kiyak G, Heydar AM, Bawaneh MK, Bezer M. Mid-length pedicle screws in posterior instrumentation of scoliosis. Asian Spine J. 2019;13(5):815–22. 31079434 10.31616/asj.2018.0177 PMC6773991

[4] Zhang JG, Wang W, Qiu GX, Wang YP, Weng XS, Xu HG. The role of preoperative pulmonary function tests in the surgical treatment of scoliosis. Spine. 2005;30(2):218–21. 15644760 10.1097/01.brs.0000150486.60895.a1

[5] N MJIJOT. Histochemical and physiological studies in idiopathic scoliosis. 1990;16(1). 2116383

[6] Gargano G, Oliva F, Migliorini F, Maffulli N. Melatonin and adolescent idiopathic scoliosis: the present evidence. Surgeon. 2022;20(6):e315–21. 34489192 10.1016/j.surge.2021.07.008

[7] Boyer J, Amin N, Taddonio R, Dozor AJ. Evidence of airway obstruction in children with idiopathic scoliosis. Chest. 1996;109(6):1532–5. 8769506 10.1378/chest.109.6.1532

[8] Farrell J, Garrido E. Effect of idiopathic thoracic scoliosis on the tracheobronchial tree. BMJ Open Respir Res. 2018;5(1). 10.1136/bmjresp-2017-000264 PMC5878681 29616140

[9] Migliorini F, Chiu WO, Scrofani R, et al. Magnetically controlled growing rods in the management of early onset scoliosis: a systematic review. J Orthop Surg Res. 2022. 10.1186/s13018-022-03200-7. 35690867 10.1186/s13018-022-03200-7 PMC9188689

[10] Fujita N, Yagi M, Michikawa T, et al. Impact of fusion for adolescent idiopathic scoliosis on lung volume measured with computed tomography. Eur Spine J. 2019;28(9):2034–41. 31177339 10.1007/s00586-019-06025-x

[11] Wen Y, Kai S, Yong-gang Z, Guo-quan Z, Tian-xiang D. Relationship between lung volume and pulmonary function in patients with adolescent idiopathic scoliosis. Clin Spine Surg A Spine Publ. 2016;29(8):E396–400. 10.1097/BSD.0000000000000161 27642778

[12] Yu CG, Grant CA, Izatt MT, et al. Change in lung volume following thoracoscopic anterior spinal fusion surgery: a 3-dimensional computed tomography investigation. Spine (Phila Pa 1976). 2017;42(12):909–16. 28609321 10.1097/BRS.0000000000001949

[13] Han C, Zhou C, Zhang H, et al. Evaluation of bone mineral density in adolescent idiopathic scoliosis using a three-dimensional finite element model: a retrospective study. J Orthop Surg Res. 2023. 10.1186/s13018-023-04413-0. 38062436 10.1186/s13018-023-04413-0 PMC10701929

[14] Edwards PD, Bull RK, Brown VS, Curtin J. Spiral CT optimization for measurement of bronchial lumen diameter using an experimental model. Br J Radiol. 2000;73(871):715–9. 11089461 10.1259/bjr.73.871.11089461

[15] Montaudon M, Berger P, de Dietrich G, et al. Assessment of airways with three-dimensional quantitative thin-section CT: in vitro and in vivo validation. Radiology. 2007;242(2):563–72. 17179398 10.1148/radiol.2422060029

[16] Zhang H, Hai Y. Surgical correction of severe scoliosis leads to changes in central airway resistance evaluated with CT-based 3D reconstruction and impulse oscillometry. J Bone Joint Surg. 2025;107(13):1506–12. 40408454 10.2106/JBJS.24.01434

[17] Zhang C, Wang H, Cao J, et al. Correction: measurement and analysis of the tracheobronchial tree in Chinese population using computed tomography. PLoS ONE. 2015. 10.1371/journal.pone.0130239. 26039717 10.1371/journal.pone.0130239 PMC4454718

[18] Laroia AT. Modern imaging of the tracheo-bronchial tree. World Journal of Radiology. 2010;2(7). 10.4329/wjr.v2.i7.237 PMC2998855 21160663

[19] Aziz R, Nachiappan C, Peter D, et al. Movement analysis of scoliotic subjects using Fastrak. 2004;91(0). 15457716

[20] Courvoisier A, Ilharreborde B, Constantinou B, Aubert B, Vialle R, Skalli W. Evaluation of a three-dimensional reconstruction method of the rib cage of mild scoliotic patients. Spine Deform. 2013;1(5):321–7. 27927387 10.1016/j.jspd.2013.07.007

[21] Gonzalez C, Ferris G, Diaz J, Fontana I, Nunez J, Marin J. Kyphoscoliotic ventilatory insufficiency: effects of long-term intermittent positive-pressure ventilation. Chest. 2003;124(3):857–62. 12970009 10.1378/chest.124.3.857

[22] LaMont LE, Jo C, Molinari S, et al. Radiographic, pulmonary, and clinical outcomes with halo gravity traction. Spine Deform. 2019;7(1):40–6. 30587319 10.1016/j.jspd.2018.06.013

[23] Mehta HP, Snyder BD, Baldassarri SR, et al. Expansion thoracoplasty improves respiratory function in a rabbit model of postnatal pulmonary hypoplasia. Spine. 2010;35(2):153–61. 20081510 10.1097/BRS.0b013e3181c4b8c7

[24] Campbell RM, Smith MD, Mayes TC, et al. The characteristics of thoracic insufficiency syndrome associated with fused ribs and congenital scoliosis. J Bone Joint Surg Am. 2003;85(3):399–408. 12637423 10.2106/00004623-200303000-00001

[25] Farrell J, Garrido E. Predicting preoperative pulmonary function in patients with thoracic adolescent idiopathic scoliosis from spinal and thoracic radiographic parameters. Eur Spine J. 2021;30(3):634–44. 32734473 10.1007/s00586-020-06552-y

[26] Lin Y, Tan H, Rong T, et al. Impact of thoracic cage dimension and geometry on cardiopulmonary function in patients with congenital scoliosis: a prospective study. Spine. 2019;44(20):1441–8. 31365514 10.1097/BRS.0000000000003178

[27] Dreimann M, Hoffmann M, Kossow K, Hitzl W, Meier O, Koller H. Scoliosis and chest cage deformity measures predicting impairments in pulmonary function: a cross-sectional study of 492 patients with scoliosis to improve the early identification of patients at risk. Spine. 2014;39(24):2024–33. 25202929 10.1097/BRS.0000000000000601

[28] Abdelaal AAM, Abd El Kafy EMAES, Elayat MSEM, Sabbahi M, Badghish MSS. Changes in pulmonary function and functional capacity in adolescents with mild idiopathic scoliosis: observational cohort study. J Int Med Res. 2017;46(1):381–91. 28661261 10.1177/0300060517715375 PMC6011275

[29] Burgos J, Hevia E, Llombart-Blanco R, et al. Pulmonary function does not improve after 10 years of posterior spinal fusion in adolescent idiopathic scoliosis: a systematic review and meta-analysis. Eur Spine J. 2025;34(5):1849–60. 40186695 10.1007/s00586-025-08831-y

[30] Wu L, Zhang XN, Wang YS, Liu YZ, Hai Y. Risk factors for pulmonary complications after posterior spinal instrumentation and fusion in the treatment of congenital scoliosis: a case-control study. BMC Musculoskelet Disord. 2019;20(1):331. 31311602 10.1186/s12891-019-2708-8 PMC6631870

[31] Qiabi M, Chagnon K, Beaupre A, Hercun J, Rakovich G. Scoliosis and bronchial obstruction. Can Respir J. 2015;22(4):206–8. 26083538 10.1155/2015/640573 PMC4530852

[32] Koumbourlis AC. Scoliosis and the respiratory system. Paediatr Respir Rev. 2006;7(2):152–60. 16765303 10.1016/j.prrv.2006.04.009

[33] Graham EJ, Lenke LG, Lowe TG, et al. Prospective pulmonary function evaluation following open thoracotomy for anterior spinal fusion in adolescent idiopathic scoliosis: Spine. 2000;25(18):2319–25. 10984783 10.1097/00007632-200009150-00009

[34] Vedantam R, Lenke LG, Bridwell KH, Haas J, Linville DA. A prospective evaluation of pulmonary function in patients with adolescent idiopathic scoliosis relative to the surgical approach used for spinal arthrodesis. Spine (Phila Pa 1976). 2000;25(1):82–90. 10647165 10.1097/00007632-200001010-00015

[35] Barrios C, Perez-Encinas C, Maruenda JI, Laguia M. Significant ventilatory functional restriction in adolescents with mild or moderate scoliosis during maximal exercise tolerance test. Spine (Phila Pa 1976). 2005;30(14):1610–5. 16025029 10.1097/01.brs.0000169447.55556.01

[36] Gadgil A, Ahmed E, Rahmatalla A, Dove J, Maffulli N. A study of the mechanical stability of scoliosis constructs using variable numbers of sublaminar wires. Eur Spine J. 2002;11(4):321–6. 12193992 10.1007/s00586-002-0473-z PMC3610475

[37] G C, V T, N MJJBJSB. Variability in Cobb angle measurements. 1996;78(2). 8666658

[38] G C, N M, V TJAOB. The validity and reliability of measurements in spinal deformities: a critical appraisal. 1992;58(2). 1632212

[39] Newton PO, Faro FD, Gollogly S, Betz RR, Lenke LG, Lowe TG. Results of preoperative pulmonary function testing of adolescents with idiopathic scoliosis. A study of six hundred and thirty-one patients. J Bone Joint Surg Am. 2005;87(9):1937–46. 16140807 10.2106/JBJS.D.02209

[40] Redding GJ, Mayer OH. Structure-respiration function relationships before and after surgical treatment of early-onset scoliosis. Clin Orthop Relat Res. 2011;469(5):1330–4. 20978878 10.1007/s11999-010-1621-0 PMC3069287

[41] Bouloussa H, Pietton R, Vergari C, Haen TX, Skalli W, Vialle R. Biplanar stereoradiography predicts pulmonary function tests in adolescent idiopathic scoliosis: a cross-sectional study. Eur Spine J. 2019;28(9):1962–9. 30895379 10.1007/s00586-019-05940-3

